# *PLOS Biology* 2015 Reviewer Thank You

**DOI:** 10.1371/journal.pbio.1002406

**Published:** 2016-02-23

The Public Library of Science (PLOS) and the *PLOS Biology* editorial team would like to warmly thank all those individuals who participated in the peer review process this past year at *PLOS Biology*. During 2015, over 1,200 reviewers from around the world helped evaluate articles submitted to the journal.

The names of our 2015 reviewers are listed in S1 Reviewer List. We are grateful to all our reviewers for sharing their expertise with *PLOS Biology* authors, and for helping us to publish a highly valued open access journal.

## Supporting Information

S1 Reviewer List Alphabetical list of 2015 *PLOS Biology* reviewers. (PDF)
