# Supplementary material for: PLOS Biology 2015 Reviewer Thank You
Source: PLoS Biol. 2016 Feb 23;14(2):e1002406. doi: 10.1371/journal.pbio.1002406 (PMC4764339; doi:10.1371/journal.pbio.1002406)
Supplement: S1 Reviewer List — (PDF) [file pbio.1002406.s001.pdf]

PLOS Biology would like to thank all those who reviewed on behalf of the journal in 2015:

Stuart Aaronson
Alejandro Aballay
Asa Abeliovich
Martin Ackermann
Christoph Adami
Richard Adams
Stein Aerts
Markus Affolter
Hervé Agaisse
Anurag Agrawal
Aneil Agrawal
Ehud Ahissar
Shawn Ahmed
Carlos Aizenman
Asifa Akhtar
John Albeck
Mattias Alenius
John Allen
Benjamin Allen
Christopher Allen
Stefano Allesina
Jose-Manuel Alonso
David Althoff
Doug Altman
Micah Altman
Sandra Amor
Hubert Amrein
Graham Anderson
Adam Anderson
Susana Andrade
Robert Angerer
Daniel Ansari
Nathalie Arhel
Luc Arnal
Gustavo Arrizabalaga
Steven Artandi
James Ashe
Kevin Ashley
Liliana Attisano
Etienne Audinat
Anthony Auerbach
Hellmut Augustin,
Nikolai Axmacher
Rava Azeredo da Silveira
Rony Azouz
Stephen Baccus
Erika Bach
Jaideep Bains
Nathalie Balaban
Lata Balakrishnan
William Balch
Robert Baldwin
Frances Balkwill
David Baltrus
Shaowen Bao
Jesse Barber
Boris Barbour
Cornelia Bargmann
Gareth Barnes
Timothy Barraclough
Jeffrey Barrett
Jeffrey Barrick
Andrew Barron
Gregory Barsh
Jiri Bartek
Nick Barton
Michelle Barton
Marlene Bartos
Uttiya Basu
Francesco Battaglia
David Baulcombe
Kayla Bayless
James Bear
Robert Beardmore
Dan Bebber
Oren Becher
Christopher Beck
Martin Beck
C. Titus Begley
Yohanns Bellaiche
Hugo Bellen
Suliann Ben Hamed
Ruha Benjamin
Steven Bensinger
Richard Benton
Sigal Ben-Yehuda
Andrea Berger
Francine Berman
Joseph Besharse
Elvire Bestion
Sven Bestmann
Carsten Beta
Stephen Beverley
Roman Biek
Holly Bik
David Bilder
Linda Birnbaum
Ewan Birney
Ingvars Birznieks
James Bisley
Linda Bisson
Ben Black
Seth Blair
Isobel Blake
Cedric Blanpain
Ran Blekhman
Brenda Bloodgood
Theodora Bloom
Luigi Boitani
Johan Bolhuis
Russell Bonduriansky
Erie Boorman
Mike Boots
Seth Bordenstein
Christine Borgman
Erich Bornberg-Bauer
G. Valentin Börner
Thomas Bosch
Kristofer Bouchard
Emmanuel Boucrot
Henri-Marc Bourbon
André Brack
Robert Bradley
Leo Brady
Amy Brand
Christiane Branlant
Michael Breakspear
Michael Brecht
Rachel Brem

Kristen Brennand
Alain Brisson
William Britt
Neil Brockdorff
Karl Broman
John Brookfield
Sam Brown
Timothy Brown
C. Titus Brown
Steven Brown
Carolyn Brown
Samantha Brugmann
John Bruno
Angus Buckling
Michael Buice
James Bull
Ed Bullmore
Sean Burgess
Scott Burgess
Tim Buschman
Frederic Bushman
Katherine Button
Daniel Butts
Christian Cajochen
Ronald Calabrese
Vince Calhoun
Stefano Calza
Andrew Cameron
Daniel Campbell-Meiklejohn
Michael Cancro
Juan Cantalapiedra
Stefano Cappa
John Carlson
Mar Carmena
Carlos Carmona-Fontaine
Sean Carroll
Rita Carsetti
Water Carson
Vivien Casagrande
Christian Casanova
Pablo Castillo
Darlene Cavalier
Patrick Cavanagh
James Cavanagh
Marie-Christine Chaboissier
Karen Chang
Brian Charlesworth
Deborah Charlesworth
Michael Chee
Julia Chekanova
Xuemei Chen
James Cherry
Dante Chialvo
Violeta Chitu
Carson Chow
Steven Chown
Peter Choyke
Pao-Tien Chuang
Jonathan Chubb
Neil Chue Hong
Thomas Clandinin
Matthew Clapham
Damon Clark
Joseph Classen
John Cleveland
Hans Clevers
Stephen Cobbold
Michael Cohen
Michael Cole
Jerome Collignon
Christos Constantinidis
Della Cook
Erik Cook
Anita Corbett
Pierre-Jean Corringer
Brian Couch
Timothy Coulson
Franck Courchamp
Charles Cox
Melissa Crawford
Sylvia Cremer
Michael Crickmore
Leroy Cronin
Elizabeth Cropper
Tyler Curiel
Jason Cyster
Cynthia Czajkowski
Abdallah Daar
Tal Dagan
Christian Dahmann
Mu-Shui Dai
Marc Dalod
Matthew Dalva
Yang Dan
Aniruddha Das
Sylvain Daujat
Jean Daunizeau
Michael David
Richard Davidson
Jamie Davies
Jonathan Davies
Ted Dawson
Scott Dawson
Troy Day
Mario de Bono
Jose de Celis
Horacio de la Iglesia
Sacco de Vries
Sue Dechenne
Michael Deem
Anthony Dell
Robert Dempski
Wu-Min Deng
Hongkui Deng
Eric Denkers
Rik Derynck
Claude Desplan
Joanne Devlin
Steven Devries
Mathew Diamond
Fred Dick
Dion Kai Dickman
Susanne Diekelmann
Stephen Diggle
George Dimopoulos
Wen-Xing Ding
Jonathan Dinman
Jose Dinneny
Marc Dionne
Rodolfo Dirzo
Jack Dixon
Andy Dobson
Chris Doe
Michael Doebeli
Tomas Dolezal
Xinzhong Dong
David Donoho
Scott Dougan
Michael Downey
Stephen Doxsey
George Dragoi
Diane Drane
Bruce Draper
David Drew

D. Allan Drummond
George Drusano
David Dubnau
Renée Duckworth
Patrick Duffy
Jeffrey Dukes
Nicholas Dulvy
Ian Dunham
Bénédicte Durand
Michael Dustin
Dieter Ebert
Michael Eck
Isaac Edery
Bruce Edgar
Scott Edwards
Gregor Eichele
Britta Eickholt
Florent Elefteriou
Olivier Elemento
Greg Elgar
Samantha Elliott
Mark Ellisman
Holger Eltzschig
Patrick Emery
Ben Emery
Barbara Engelhardt
Per Ericson
Monique Ernst
Catherine Etchebest
Phil Evans
Jonathan Ewbank
Adam Eyre-Walker
Thomas Ezard
Robert Fairclough
Daniele Fanelli
Eric Fearon
Michael Federle
Daniel Feldman
Stuart Ferguson
Israel Fernandez
Rafael Fernandez-Chacon
Rodrigo Fernandez-Gonzalez
Matthew Ferrari
Régis Ferrière
Greg Field
Zoe Finkel
Rick Firtel
Urs Fischer
Wolfgang Fischle
Simon Fisher
Yonatan Fishman
Nicholas Fisk
J. Ross Fitzgerald
John Flanagan
Damien Fordham
Bryan Foster
Kevin Foster
Russell Foster
Mirko Francesconi
Steven Frank
David Freedman
Matthew Freeman
Eckhard Friauf
Scott Frickel
Thomas Friedrich
Elaine Fuchs
Toni Gabaldón
Harrison Gabel
Bernhard Gaese
Jean-Michel Gaillard
Albert Galaburda
Mary Galinski
Marco Gallio
Karunesh Ganguly
Ian Ganley
Olga Garaschuk
Mariano Garcia-Blanco
Andy Gardner
Timothy Gardner
Richard Gardner
Preston Garraghty
Audrey Gasch
Anne Gatignol
Thierry Gaude
Po-Wu Gean
Lisa Genzel
Holger Gerhardt
Asif Ghazanfar
Wade Gibson
Jack Gilbert
Wendy Gilbert
Darren Gilmour
Anne-Lise Giraud
Bojana Gligorijevic
Marcia Goldberg
David Goldston
Jesus Gomez-Gardenes
Venugopala Reddy Gonehal
Angel Goñi-Moreno
Eva Gonzalez-Suarez
Geoffrey Goodhill
Miriam Goodman
Vera Gorbunova
Isabel Gordo
Jeff Gore
Chris Gorgolewski
David Gosser
Jean Gotman
Berthold Gottgens
Jacqueline Gottlieb
Bruno Goud
Julian Gough
Kathleen Gould
Francesca Granucci
Nicholas Grassly
Dana Graves
Joseph Graves
Giuseppe Graziano
E. Peter Greenberg
Savraj Grewal
Leslie Griffith
Gillian Griffiths
Erwin Grill
Nick Grishin
Yael Grosjean
Lou Gross
Thilo Gross
Joachim Gross
Kay Grunewald
Calin Guet
Yan Guo
Sunetra Gupta
Jessica Gurevitch
Eric Haag
James Haber
Matthew Hahn
Norbert Hajos
Steve Halford
Michael Hall
Steven Hallam
Benedikt Hallgrímsson
Weiping Han
Inga Hänelt
Ileana Hanganu-Opatz

Jacob Hanna
Matthew Hansen
Bill Hansson
Nicholas Harden
Michael Harfoot
Laura Harrington
Reuben Harris
Ron Harris-Warrick
Kieran Harvey
Alan Hastings
Michael Häusser
James Haxby
Cole Haynes
Megan Head
Tyson Hedrick
Ruth Heidelberger
Andreas Hejnol
Siegfried Hekimi
Johannes Hell
Michael Hemann
Charlotte Hemelrijk
Scott Hensley
Andrew Herr
Christoph Herrmann
Ryan Hibbs
Michael Hickey
Tetsuya Higashiyama
Julian Higgins
Jonathan Higgins
Kent Hill
Anne Hinderliter
Yasushi Hiraoka
Karen Hirschi
Chris Hittinger
Oliver Hobert
Andreas Hochwagen
David Hockenbery
Eva Hoffmann
Peter Hohenstein
V. Michael Holers
Daniel Hollander
Edward Holmes
Todd Holmes
Charles Holmes
Anthony Holmes
Robert Holmgren
Robert Holt
Scott Hooper
Thomas Hope
Alan Horsager
Nancy Horton
Alan Horwitz
Peter Hotez
Asbjørn Hróbjartsson
Jian Hua
Sui Huang
Bernhard Hube
Kathryn Huff
Richard Huganir
John Huguenard
Thomas Hummel
Laurence Hurst
Ed Hurt
Benjamin Hutchinson
Anna Huttenlocher
Marko Hyytiäinen
Michael Ibbotson
Zoya Ignatova
Beat Imhof
Jean-Luc Imler
Nicholas Ingolia
Gareth Inman
Robert Insall
Darren Irwin
Antonio Jacinto
Matthew Jacobson
Lasse Jakobsen
David James
Kim Janda
Eckhard Jankowsky
Heinrich Jasper
Albert Jeltsch
Michael Jennions
Ole Jensen
Chris Jiggins
Tian Jin
Naihe Jing
Gareth John
Alexander Johnson
Olivier Joly
David Jones
William Kaelin
Pascal Kaeser
Jochen Kaiser
Daniel Kalderon
Suzan Kalisz
Ryota Kanai
Maya Kansara
Pankaj Kapahi
Jack Kaplan
Zaven Kaprielian
Mariusz Karbowski
Katrin Karbstein
François Karch
Natasha Karp
S. Ananth Karumanchi
Anna Kashina
Kerstin Kaufmann
Daniel Kearns
Jack Keene
Lukas Keller
Steve Kelling
Eric Kemen
Caleb Kemere
Steven Kennerley
René Ketting
Sepidah Khorasanizadeh
Chaitan Khosla
Thomas Kidd
Susan Kidson
Timothy Kieffer
Daniel Kiehart
A. Marm Kilpatrick
Do-Hyung Kim
Jeansok Kim
Stuart Kim
Rebecca Kimball
Jonathan Kimmelman
Akatsuki Kimura
Aaron King
Sue Kinn
Tomas Kirchhausen
Wolfgang Kirchner
Thomas Kirkwood
Roy Kishony
Axel Kleidon
David Kleinfeld
Miriam Klein-Flügge
Jens Kleinjung
Richard Kliman
Wolfgang Klimesch
Sandra Knapp
Laura Knoll
Bartha Knoppers

Katia Koelle
Roberto Kolter
Alexey Kondrashov
Mei Kong
Genevieve Konopka
Maarten Koornneef
Ryszard Korona
Martin Korte
Achim Kramer
Nina Kraus
Gabriel Kreiman
Beth Krizek
Daniel Kronauer
Paul Kubes
John Kubie
Ulrich Kück
Patricia Kuhl
Rohit Kulkarni
Dimitri Kullmann
Rohini Kuner
Edo Kussell
David Kwiatkowski
Denis Lafontaine
Peter Lakatos
Amaury Lambert
Gert Lanckriet
David Lane
Ralph Langen
Morgan Langille
Michael Lanzer
Hilmar Lapp
Pekka Lappalainen
Eicke Latz
Michael Laub
Simon Laughlin
William Laurance
Karen Laurie
Adam Lauring
Julie Law
Beth Lazazzera
Matthew Lazzara
Brian Lazzaro
Nicolas Le Novère
Karine Le Roch
Sang Eun Lee
Tzumin Lee
Cheng-Yu Lee
Seok-Yong Lee
Rob Leech
Ruth Lehmann
Brian Lehmann
Ed Lein
Anna Lenard
David Lentink
Fred Lenz
Edward Leof
David Leopold
Sarah Lester
Johan Leveau
Michael Levin
Emmanuel Levy
Alexsandra Lewandowska
Jason Lewis
Ian Lewkowich
Tom Libby
Stephen Liberles
Michael Lichten
Xiaorong Lin
Xuefeng Ling
Dirk Linke
Timothy Linksvayer
Tom Little
Haoping Liu
James Lloyd-Smith
Stanley Lo
Jan Lohmann
Kirk Lohmueller
Joseph Loparo
Daniel López
Anton Lord
Zhenkun Lou
Thomas Lovejoy
Guillermina Lozano
Robert Lucas
Lawrence Lum
Kunxin Luo
Andrei Lupas
Bernhard Luscher
Jared Lyle
Hong Ma
Richard Maas
Georgina Mace
Laura Machesky
Wendy Macklin
Malcolm Macleod
Pierre Magistretti
Ari Pekka Mahonen
Joel Mainland
William Mair
Bonaventura Majolo
Alexei Maklakov
Harmit Malik
Jim Manfredi
Richard Mann
Gabriel Marais
Christian Margot
Julien Marie
Alberto Marina
Hendrik Marks
Rogier Mars
Joseph Marsh
James Marshall
Dustin Marshall
Adele Marston
Tom Martin
Graham Martin
Julio Martinez
Micaela Martinez-Bakker
Adam Martiny
Marcello Massimini
Naoki Masuda
Daniel Matute
Marjori Matzke
Robert May
Kim McAllister
Kevin McCann
Sheila McCormick
James McGaugh
Brian McGill
Elizabeth McGraw
Aoife McLysaght
Kelly McNagny
Mitch McVey
David Meek
Johanna Meijer
Richard Meisel
Markus Meissner
Giuseppe Melacini
Juan Melero-Martin
Gregory Melikian
Jack Mellor
Hugo Merchant
Alex Meredith
Christien Merrifield

Jessica Metcalf
Martin Meyer
Blake Meyers
Thandi Mgwebi
Stephen Michnick
Nicole Mideo
Tam Mignot
Irene Miguel-Aliaga
Marco Milan
Jocelyn Millar
Samuel Miller
Robert Miller
Jordan Miller
Chuck Miller
Baruch Minke
Eric Miska
Kevin Mitchell
James Mitchell
Yasushi Miyashita
Kenneth Moberg
Edward Mocarski
Mauro Modesti
Hervé Moine
Christine Moissl-Eichinger
Andreas Möller
Denise Monack
Craig Montell
Frits Mooi
Charles Moran
Veronica Morea
David Moreira
Phil Morgan
Levi Morran
Lynn Morris
Maria Concetta Morrone
J. Bruce Morton
Michael Moseley
Eric Moss
Maria Mota
Anthony Movshon
Cheryl Moyer
Sean Mullen
Daniel Müller
William Muller
Ralph Müller
Peter Mumby
Michael Murphy
Peter Murray
Andrew Murray
Christopher Murray
John Murray
Michael Mwangi
Karim Nader
Laszlo Nagy
Shinichi Nakagawa
Yoshinobu Nakanishi
Eiji Nambara
Jordi Navarra
Anne Negre-Salvayre
Israel Nelken
Celeste Nelson
David Nemazee
Anje-Margriet Neutel
Richard Neve
Dianne Newman
Christopher Nicchitta
Benjamin Nichols
Christof Niehrs
Rasmus Nielsen
Meredith Niles
Ove Nilsson
Zachary Nimchuck
Lee Niswander
Michael Nitabach
B. Tracy Nixon
Suzanne Noble
Mohamed Noor
Uta Noppeney
Sergei Noskov
Peter Novick
Rachel Nugent
Ruth Nussinov
Scott Oakes
Jon Oatley
Darren Obbard
Philipp Oberdoerffer
Andrew Oberst
Howard Ochman
Daniel O'Connor
Michael O'Connor
Paul Ode
John O'Doherty
Yasushi Okada
Bjorn Olsen
Michael Olson
Chitose Oneyama
Christine Orengo
Walter Orenstein
Harry Orr
Terry Orr-Weaver
Sarah Otto
Craig Packer
Michele Pagano
Rod Page
Leo Pallanck
Arthur Palmer
Bernhard Palsson
Søren Paludan
Zhuo-Hua Pan
Jason Papin
Olivier Pardo
Linda Partridge
Josef Parvizi
Mercedes Pascual
R. Jeroen Pasterkamp
Nipam Patel
Samraat Pawar
Rony Paz
Jean Peccoud
Laurence Pelletier
David Penny
Nathalie Percie du Sert
Alan Perelson
Norbert Perrimon
Andreas Peschel
Luiz Pessoa
Carl Petersen
Townsend Peterson
Gregory Petsko
Roberto Pezza
John Phillips
Martin Pickering
Richard Pickersgill
Christopher Pierce
Jonathan Pierce-Shimomura
Daniele Piomelli
Virginia Pitzer
Timothée Poisot
Jean-Baptiste Poline
Jessica Polka
Michael Pollak
Magdalini Polymenidou
Anthony Poole
Richard Possemato

Hugh Possingham
S. Steven Potter
Evan Powers
Immo Prinz
Peter Pryciak
Kathleen Pryer
Bali Pulendran
Louise Purton
Bin-Zhi Qian
Britta Qualmann
Wim Quax
Alfredo Quinones-Hinojosa
Alan Rabinowitz
David Raible
Paul Rainey
Ayyalusamy Ramamoorthy
Franck Ramus
Troy Randall
Chad Rappleye
Matthew Rasband
Peter Ratcliffe
Kodi Ravichandran
Anandasankar Ray
Andrew Read
Mark Rebeiz
Rosemary Redfield
A. David Redish
Jason Reed
Douglas Rees
Roland Regoes
Yvonne Reid
Anna Renwick
Diego Restrepo
Kim Rewitz
Claude-Agnes Reynaud
Gal Ribak
Thomas Richards
Robert Ricklefs
Sebastien Rigali
Claire Rind
Deborah Roach
Silke Robatzek
Paul Robbins
Edwin Robertson
Mel Robertson
Eduardo Rocha
Tristan Rodriguez
Robert Roeder
Pieter Roelfsema
Eleanor Rogan
Dragana Rogulja
Rajat Rohatgi
Antonis Rokas
Martin Rolfs
Ranulfo Romo
Maria Ros
Michael Rose
Jeffrey Rosen
Helene Rosenberg
Robert Roskoski
Robert Rottapel
Sarah Rowland-Jones
Peter Roy
Michele Rucci
Christian Ruff
Eytan Ruppin
Matthew Rushworth
Paul Russell
Andrew Russell
Vanessa Ruta
Jared Rutter
Guy Rutter
Timothy Ryan
Robert Sablowski
Kalie Sacco
Timothy Sackton
Alan Saghatelian
Lisa Saksida
Aman Saleem
Kourosh Salehi-Ashtiani
Daniel Salmon
Tim Salomons
Aziz Sancar
Jean-Charles Sanchez
Jürgen Sandkühler
Sanjay Sane
Owen Sansom
Elizabeth Sattely
Herbert Sauro
Nick Savill
Andreas Schaefer
Jeffrey Schall
Jorn Scharlemann
Ben Scheres
Leonhard Schilbach
David Schimel
Alejandro Schinder
Enrico Schleiff
Patrick Schloss
Sandra Schmid
Marc Schmidt-Supprian
Lars Schmitz
David Schneider
Elad Schneidman
Jon Scholey
Gunnar Schotta
Julian Schroeder
Patrick Schultz
Michael Schurr
Ivan Schwab
Robert Schwabe
Cornelius Schwarz
Luca Scorrano
Nava Segev
Julie Segre
Amita Sehgal
Terrence Sejnowski
Gregg Semenza
Mihaela Serpe
Ben Seymour
David Shackelford
Reza Shadmehr
Premal Shah
Kwang-Tsao Shao
Robert Shapley
Phillip Sharp
Michael Sheehan
Osamu Shimmi
Alexander Shingleton
David Shore
Spencer Shorte
Oren Shriki
Yousheng Shu
Joshua Shulman
Susanne Shultz
Derek Sieburth
Shai Silberberg
Bruno Silva-Santos
Pamela Silver
Daniele Silvestro
Caroline Simard
Hans-Uwe Simon
Martine Simonelig
Michael Simons

Uri Simonsohn
Laura Sim-Selley
Saurabh Sinha
Haruhiko Siomi
Jon Slate
Dirk-Jan Slotboom
Richard Smith
Evan Snitkin
Michael Soares
Alexander Sobolevsky
Vikaas Sohal
David Soll
Mark Sorrells
Wayne Sossin
Ileana Soto
Nuno Sousa
Rosangela Sozzani
Roger Spealman
Maria Spies
Olaf Sporns
Robert Stackman
Tanja Stadler
Phillip Staniczenko
Kenneth Stanley
Jorg Stelling
Gudrun Stenbeck
Patrick Stephens
David Stern
Robert Stickgold
Ann Stock
Hugo Stocker
Peter Stoilov
Caleb Strait
Carly Strasser
Joan Strassmann
Jeffrey Streelman
Eric Strieter
Rob Striker
Nathalie Stroeymeyt
Gary Struhl
Grace Stutzmann
Dong-Ming Su
Yi-Hsien Su
Greg Suh
Alexander Suh
Seirian Sumner
Carolyn Susuki
Katalin Susztak
Granger Sutton
Roger Sutton
Harumi Suzuki
Jason Swedlow
W. Edward Swords
Lorraine Symington
Eörs Szathmáry
Larry Taber
Paul Taghert
Atsuko Takashima
Tadaomi Takenawa
William Talbot
Mark Tanaka
Minoru Tanaka
Guy Tanentzapf
Dan Tawfik
Terrie Taylor
Susan Taylor
Derek Taylor
Aurelio Teleman
Derk Ten Berge
Carel Ten Cate
Adrian Teo
Marc Therrien
Denis Thieffry
Barry Thompson
Stefan Thor
Andrew Thorburn
Janet Thornton
Sally Thurston
Gregor Thut
Paul Tiesinga
Ian Tizard
Sokol Todi
Jared Toettcher
Susumu Tonegawa
Frank Tong
Miguel Torres
Jan Traas
Elizabeth Tran
Justin Travis
Bebhinn Treanor
Tom Tregenza
Mathias Treier
Nicolas Tricaud
Ralph Tripp
Patrice Trouiller
Li-Huei Tsai
Christian Tschudi
Burkhard Tümmler
Jerrold Turner
James Turner
Lucina Uddin
Tobias Uller
Taishi Umezawa
Nathaniel Urban
Aart van Bel
Josh van Buskirk
Kathleen van Craenenbroeck
Raoul van Damme
Marcel van der Heijden
Bart van der Worp
Jacco van Rheenen
Jos van Strijp
Bruno van Swinderen
Vicki Vance
Eric Vander Wal
Pierre Vanderhaeghen
Wim Vanduffel
David Vaux
Dimitrios Vavylonis
Kartik Venkatachalam
J. Craig Venter
Kevin Verstrepen
Cecile Viboud
Beatriz Vicoso
Jonathan D. Victor
Liisa Viikari
Timothy Vines
Todd Vision
Octavian Voiculescu
Arndt von Haeseler
Eric-Jan Wagenmakers
Tor Wager
Günter Wagner
Erwin Wagner
Andreas Wagner
Matthew Waldor
Neil Walker
David Wallach
John Wallingford
Peter Walter
Xiaochen Wang
Ming-Bo Wang
Weidong Wang
Stephen Ward

Robert Warner
Andrew Waskiewicz
Samuel Wasser
Marta Wayne
Wenyi Wei
Cornelis Weijer
Dolf Weijers
Jean-Claude Weill
David Weinberg
William Weiss
Nathan Weisz
Dave Wemmer
Johan Wessberg
Stuart West
Chris Westlake
Helen White-Cooper
Malcolm Whitman
Katja Wiech
John Wilbanks
Siouxsie Wiles
Dagmar Wilhelm
Claus Wilke
Robert Wilkinson
David Williams
Christopher Wilmers
James Wilsdon
David Wilson
Melissa Wilson Sayres
G. Elliott Wimmer
D. Michael Winter
Dyann Wirth
Richard Witter
Martin Wojtowicz
Jochen Wolf
Kenneth Wolfe
Jonathan Wolpaw
Thilo Womelsdorf
Mark Woolhouse
Kevin Woollard
Henry Wortis
Gregory Wray
Chris Wright
Louisa Wu
Long-Jun Wu
F. Gregory Wulczyn
Yang Xiang
Dajun Xing
Daisuke Yamamoto
Itai Yanai
Humphrey Yao
George Yap
Michael Yassa
Dag Yasui
Andrew Yates
John Yin
Michael Young
Yossi Yovel
Michael Yudell
Gabriel Yvon-Durocher
Anthony Zador
David Zarkower
Jianzhi Zhang
Qing Zhang
Mei Zhen
Bin Zheng
Ming-Ming Zhou
Ronghua Zhuge
Min Zhuo
Manuel Zimmer
Joshua Zimmerberg
Karen Zito
Guy Ziv
Marta Zlatic
Berislav Zlokovic
Davide Zoccolan
David Zusman
Arturo Zychlinsky
